# Evidence-Based Integrative Health Care to Promote Quality of Life in Older Adults With Cancer

**DOI:** 10.1093/geroni/igab046.2325

**Published:** 2021-12-17

**Authors:** Robin Majeski, Delia Chiaramonte

**Affiliations:** 1 University of Maryland, Baltimore County, Catonsville, Maryland, United States; 2 Greater Baltimore Medical Center, Towson, Maryland, United States

## Abstract

Cancer disproportionately affects older adults and presents significant challenges to patients’ quality of life. Use of complementary medicine is increasing among older adults with cancer and these modalities have the potential for both benefit and harm. Thus, it is important that health care professionals are knowledgeable about the evidence-supported benefits and risks of complementary and integrative health approaches in the care of older adults with cancer. Integrative cancer care provides a comprehensive approach to reducing symptom burden in patients suffering with cancer symptoms and side effects of cancer treatment. Symptoms such as pain, fatigue, nausea, sleep disturbance, mood disorder, perceived stress, and reduced quality of life are common in this population.This session will discuss an evidence-based integrative approach to cancer care which incorporates both pharmacologic and non-pharmacologic modalities to decrease symptom burden, enhance patient well-being, and improve quality of life. Non-pharmacologic modalities used in the integrative approach to care will be described and relevant evidence for risks, benefits and indications will be presented. Case studies will be discussed to demonstrate the integration of these techniques into conventional western medical treatment plans for older adults with cancer. Diversity and inclusion issues relevant to integrative medicine for underserved cancer patients will be addressed, as well as recommendations for future research to expand access of underserved populations to evidence-supported integrative cancer care. A resource list will be provided to participants.

